# Positive mRNA Translational Control in Germ Cells by Initiation Factor Selectivity

**DOI:** 10.1155/2015/327963

**Published:** 2015-08-19

**Authors:** Andrew J. Friday, Brett D. Keiper

**Affiliations:** Department of Biochemistry and Molecular Biology, Brody School of Medicine, East Carolina University, Greenville, NC 27834, USA

## Abstract

Ultimately, the production of new proteins in undetermined cells pushes them to new fates. Other proteins hold a stem cell in a mode of self-renewal. In germ cells, these decision-making proteins are produced largely from translational control of preexisting mRNAs. To date, all of the regulation has been attributed to RNA binding proteins (RBPs) that repress mRNAs in many models of germ cell development (*Drosophila*, mouse, *C. elegans*, and *Xenopus*). In this review, we focus on the selective, positive function of translation initiation factors eIF4E and eIF4G, which recruit mRNAs to ribosomes upon derepression. Evidence now shows that the two events are not separate but rather are coordinated through composite complexes of repressors and germ cell isoforms of eIF4 factors. Strikingly, the initiation factor isoforms are themselves mRNA selective. The mRNP complexes of translation factors and RBPs are built on specific populations of mRNAs to prime them for subsequent translation initiation. Simple rearrangement of the partners causes a dormant mRNP to become synthetically active in germ cells when and where they are required to support gametogenesis.

## 1. mRNA Translation Initiation Activity Matters for Cell Fate

**Figure 1:**
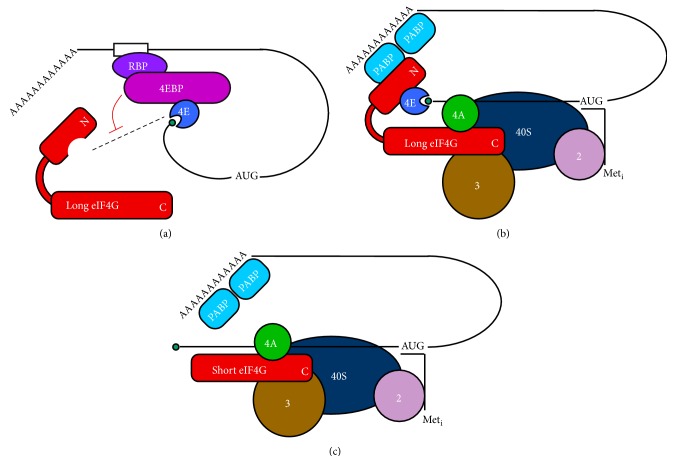
Models of mRNA translational repression and translation initiation complexes. (a) mRNAs are translationally repressed by RBPs that bind sequence recognition motifs in the 3′UTR. Protein-protein interactions with 4EBP-eIF4E-mRNA form stable mRNP complexes that inhibit the recruitment of eIF4E-bound mRNA to eIF4G, eIF4A (an mRNA helicase), and the ribosome. (b) Model of cap-dependent translation initiation utilizing the cap-binding protein eIF4E. Cap-bound mRNAs are recruited to the 40S ribosomal subunit by association with eIF4G and PABP. Association with eIF2 and eIF3 completes the 48S preinitiation complex. (c) Model of cap-independent translation. The “short” isoform of eIF4G lacking an eIF4E-binding domain is still capable of recruiting mRNA to the 40S ribosomal subunit with eIFs by binding directly to the 5′UTR.

Stem cell self-renewal and differentiation programs in the germline depend on gene expression largely regulated at the level of protein synthesis. The central dogma of gene expression states that information moves from DNA to RNA to protein, where mRNA translation represents a final step in protein expression. Regulation of mRNA translation is important for the differentiation of stem cells into terminal cell types, as they dictate the identity of the resulting new cell type. In a somatic example, myocyte differentiation eventually requires significant synthesis of myosin and actin. During neurogenesis, blast cells both extend processes as a function of cell adhesion molecules and express ion channels that make them electrically active. Germline stem cells (GSCs) seem to make the greatest use of protein synthetic regulation to guide their differentiation into sperm and eggs. Dysregulation of these protein synthetic events (at any level and in any “plastic” cell) can lead to aberrant developmental defects including infertility, birth defects, and cancers. Proteins in the mRNA translation initiation complex (called “eIFs,” eukaryotic initiation factors) play a key role in beginning the protein synthesis that ultimately completes such cell fate decisions. Among these factors, eIF4E, which binds the mRNA 7-methyl-GTP cap, and eIF4G, which scaffolds the cap, poly(A) tail, and eIF2, eIF3, eIF4A, and the ribosome together, have a critical role in recruiting mRNA to the protein synthetic machinery (Figure 1(b)). eIF4GI, for instance, is overexpressed in breast and lung cancers and enhances the translation of mRNAs that are involved in survival (Hif-1a, VEGF), cell signaling (cadherins), and DNA damage repair (p53, p53-BP1, and PARP) [1–6]. High levels of the cap-binding protein eIF4E promote cell growth and proliferation in cancers including ovarian, esophageal, breast, thyroid, and prostate cancers, as well as leukemias [7–12]. Association of eIF4E with eIF4G by phosphorylation of 4EBP is under the regulation of the kinase mTOR [13–15]. This signaling event has been shown to play a role in the fetal development of distal lung epithelium during perinatal stages of gestation [16]. As in fetal development, dysregulation of protein expression in germ cells has dire consequences for gametogenesis. Protein dysregulation contributes to infertility in 12–15% of couples worldwide [17]. Many mRNAs stored in germ cells have short poly(A) tails and can be activated in response to poly(A) elongation [18, 19]. RNA binding proteins (RBPs), such as DAZL family members, recruit poly(A)-binding protein (PABP) and initiation factors to bind to the mRNA, thus promoting translation initiation. Mutations in DAZL RBPs expressed in prenatal and postnatal germ cells result in infertility and sterility [20, 21]. Given the numerous circumstantial relationships between mRNA translational control, stem cells, gametogenesis, and cancers, it seems prudent to look for similar mechanisms in each.

## 2. The Protein Synthetic Needs of Germline Stem Cells (GSCs)

How likely is it that mRNA initiation mechanisms are governing germ cell life, where cell fates are just being established, given their roles in oncogenesis, where established fates are overturned? We operationally define stem cells as a population with the unique ability to be maintained by a self-renewal program, yet equally capable of differentiating into a spectrum of functional cellular lineages. Somatic stem cells are present at low abundance throughout tissues or reside in segregated niches [22]. Their main function is to replenish localized cells following injury or disease and during normal growth. Germline stem cells (GSCs), however, possess the unique ability to differentiate and enter meiosis to form single-celled, autonomous gametes for transgenerational success of the species. They are the only truly immortal lineage of cells in higher eukaryotes, and their main role is to transmit genetic information to future progeny. GSCs rely on* de novo* protein synthesis to drive their potential cell fates that include proliferation/self-renewal, differentiation, or apoptosis. Proliferation guarantees the sustained production of gamete progenitors and a renewable pool of GSCs. Differentiation programs allow specialized morphing of GSCs into viable sperm and egg fates. Each program requires novel proteins for meiosis that condense, replicate, recombine, resolve, and segregate the chromosomal pool. At the same time, cytoplasmic components to supply the fertilized egg are synthesized or deposited in female germ cells (oocytes) or stripped away from male germ cells (spermatocytes). Lastly, proteins that allow each gamete to recognize, cooperate with, and fuse with the other are synthesized during maturation. Apoptosis is also a germ cell fate previously underappreciated for its contribution to gamete development and is surprisingly active in the germline. In many organisms, nurse cells are derived from germ cells that commit themselves to apoptosis in order to contribute cytoplasmic mRNAs, proteins, organelles, and other components to their sibling germ cells that mature into gametes [23, 24].

## 3. Germ Cells Use mRNA Repression/Activation to Control the Timing for Introducing New Functions

Translational recruitment of mRNAs for protein synthesis in germ cells is crucial during a period of transcriptional silencing. Early oocytes accumulate and store maternal mRNAs and RBPs to be utilized for* de novo* protein synthesis during critical periods of development in oogenesis and embryogenesis. During* C. elegans* oogenesis, for example, chromosome condensation causes transcriptional silencing that remains in effect until the 2-cell embryo [25, 26]. Genes encoding proteins required during this period are transcribed early in germ cell life and the mRNAs stored in ribonucleoprotein particles (mRNPs) for later use. Germ cells also accumulate translational machinery, some of it unique to the germline. Stored mRNPs and translation factors provide the developing gametes and embryos with new proteins necessary for development and maturation [27–29]. Mouse oocytes accumulate maternal mRNA and proteins during a growth phase of nearly two weeks that are similarly required during ovulation, fertilization, and early embryonic divisions. However, the embryonic genome is only partially available at the 2-cell stage and embryos rely heavily on stored mRNAs for translation until the 8-cell stage [30].* Xenopus* embryos are perhaps the most extreme, as they remain transcriptionally silent through the first 12 divisions (4000 cells) at which point their cell cycle slows down and transcription resumes [31]. Inability to translate stored mRNAs would cause both defective gametes and embryonic lethality [27, 28].

One prevalent mechanism for regulating the spatial and temporal utilization of mRNAs in development uses RBP complexes that bind to recognition motifs in mRNA 3′UTRs to repress their translation. Dissociation of these RBPs is thought to cause derepression of mRNPs and allow for new protein synthesis to occur where and when it is needed in germ cell progression. For example, highly conserved (from yeast to mammals and plants) PUF proteins have been implicated in maintaining mitotic proliferation and the self-renewal of GSCs [32, 33]. Loss of PUF protein function results in the failure of GSC asymmetric divisions and promotes their precocious differentiation as well as germ cell tumors [34]. In the* C. elegans* germline, an elegantly complex and progressive series of translational control events govern nearly every step in the transition of GSCs from mitosis into meiosis and through maturation. Two proteins integral to these regulatory mechanisms are the PUF homologs FBF-1 and FBF-2. The choice between sperm and oocyte differentiation is also decided by mRNA translational control [35, 36]. In larval gonads, the sperm/oocyte switch is set to “sperm on” due to the repression of* tra-2* mRNA by the RBP complex GLD-1/FOG-2. In adult hermaphrodites, however, GLP-1/Notch signaling promotes the FBF/NOS-3 complex repression of* fem-3* mRNA, which switches new germ cells to “sperm off” and promotes differentiation into oocytes. The GLP-1/Notch signal itself is a product of translational control. Inverse translational repression of* gld-1* (promeiotic) and* glp-1* (promitotic) mRNAs regulates the GSCs transition from mitosis to meiosis. The delicate balance of this mRNA regulation is such that ectopic expression of GLP-1 results in unabated germ cell mitosis and germline tumor formation with little or no germ cell differentiation [37]. Furthermore, partial* gld-1* loss of function abolishes oogenesis and germ cells arrest in pachytene. More extreme loss of* gld-1* (null) results in pachytene-stalled germ cells that return to the mitotic cell cycle and form germline tumors [38]. In* Drosophila* GSCs, a Vasa (eIF4A-like helicase) eIF5B complex exerts mRNA translational control that involves repression and subsequent activation to restrict the “renewal” cell fate as well. Vasa (−/−) females exhibit egg chambers with undifferentiated nurse cells and oocyte tumors, demonstrating again a pivotal role for translational regulation in GSC fate [39, 40]. The canonical translation factor eIF4A also has a vital role in maintaining GSC self-renewal by inhibiting the function of BAM in an mRNP complex [41]. Toward the end of germ cell development, oocytes and spermatocytes rely on translational control for proper growth and differentiation. In arrested stage VI* Xenopus* oocytes, progesterone signaling induces strong* cyclin B* and* c-mos* (serine-threonine kinase) mRNA translation. New synthesis of these proteins activates Cdc-2-Cyclin B kinase to promote cell cycle resumption and meiotic maturation [42, 43]. In late stage arrested* C. elegans* oocytes, OMA-1 and OMA-2 (redundant RBPs) are required for progression through meiotic prophase I [44]. At later points, the OMA-1/LIN-41 RNP is an essential regulator of oocyte growth and maturation through translational repression of target mRNAs [45, 46]. Recent data suggests that an OMA protein inhibits* wee-1.3* (Myt1 homolog) mRNA translation, preventing the inactivation of CDC-25.1 and subsequently CDK-1. This phosphatase/kinase cascade promotes cell cycle resumption in maturing oocytes [44, 47]. At many junctures during gametogenesis, translational regulation of germ cell mRNAs plays an integral role in meiotic cell cycle checkpoints for development of viable and mature gametes.

## 4. mRNA Recruitment for New Protein Synthesis

As mRNAs become derepressed, the translation initiation machinery recognizes and recruits them to ribosomes to drive new protein synthesis required for development. Growing evidence suggests that the repression events are coordinated with derepression events to allow for precisely timed* de novo* synthesis of novel proteins as required. The limitation in a cell's ability to utilize derepressed mRNAs is mobilizing each mRNA to ribosomes via a translation initiation complex. For most mRNAs, mobilization begins with eukaryotic initiation factor 4E (eIF4E) binding to the mRNA 7-methylguanosine 5′ cap structure for recruitment to the 40S ribosomal subunit [48, 49]. eIF4E-bound mRNAs associate with the eIF4G scaffolding protein and the eIF4A helicase to form a productive eIF4 complex. Recruitment of eIF4-bound mRNAs to 40S subunit occurs by the synergistic function of the eIF4 proteins and PABPs bound to the poly(A) tail [50] (Figure 1(b)). eIF4G coordinates eIF4E and PABP and it binds mRNA to promote the assembly of a “closed loop” circular mRNP. Circularization is proposed to facilitate the recycling and reinitiation of posttermination ribosomes, thus increasing the mRNA's translational efficiency [51]. PABP also helps recycle 60S ribosomal subunits to the preinitiation complex [52]. Within these mechanisms, eIF4E and eIF4G play key roles in regulating mRNA translational control for cap-dependent protein synthesis.

Both eIF4E and eIF4G are highly conserved across species (yeast to human), and each has been implicated in various cell fate decisions (see Section 1). Developmental cell fates seem to respond to the types of eIF4E and eIF4G isoforms, or indeed unique germ cell isoforms, represented in a given cell. For example, three eIF4E proteins have been characterized in mammals, five in* C. elegans*, three in* Xenopus*, three in plants, three in zebrafish, and eight in* Drosophila* [53–57]. Several are unique to or are the predominant form in germ cells [54, 56, 58–61]. In* Drosophila*, unique eIF4Es regulate the translation of* oskar* mRNA, which is necessary for embryonic posterior patterning and germ cell formation [57, 62]. Isoforms eIF4E-1 and eIF4E-3 are required for meiotic stages of spermatogenesis [61]. In* Xenopus* oogenesis, eIF4E1b has been identified in an mRNP complex responsible for the repression of meiotic maturation in early stage oocytes [59]. This protein associates with a novel 4EBP called eIF4E-T that transports and sequesters the cap-binding protein. In* C. elegans* eIF4E isoforms have been shown to regulate cell fate decisions not only in the germline (IFE-1: sperm and oocyte maturation; IFE-2: meiotic recombination; IFE-3: sperm to oocyte switch), but also in somatic tissue (IFE-2: animal longevity, IFE-4: muscle and neuron development) [54, 63–68] (Keiper, unpublished).

Likewise, eIF4G isoforms (IFG-1 p170 and p130) have also been shown to carry out distinctly differing roles in germ cell fate decisions. IFG-1 p170 is the integral scaffolding protein in the m^7^G-binding translation initiation complex and supports cap-dependent protein synthesis. This “long” eIF4G form binds eIF4Es (IFEs) and promotes germ cell proliferation and oogenic differentiation [64]. Long eIF4G was also shown to be essential for translation of cell cycle mRNAs like* c-mos* in* Xenopus* oocytes [69]. Cleavage of eIF4G to a “short” form supports only the cap-independent initiation mechanism for protein synthesis. Over 70% of protein synthetic capacity remains intact in “cap-independent” oocytes, but their capacity to undergo meiotic maturation (cell cycle progression) in response to progesterone is lost [69, 70]. Similarly, the naturally occurring* C. elegans* IFG-1 p130 (short form) that lacks the eIF4E-binding domain supports the cap-independent initiation of housekeeping mRNAs and stress related mRNAs during germ cell apoptosis [71, 72]. The cap-independent mechanism was originally discovered for viral mRNAs that become translated more efficiently when eIF4G is cleaved [73]. IFG p130 cap-independent translation, for example, of* Hsp70* and* Bcl-2* mRNAs, provides germ cells that are in distress (perhaps from meiotic DNA recombination gone wrong) with an opportunity to recover and survive during a resolution period [72]. Damage that is too severe also uses cap-independent synthesis to initiate programmed cell death, or apoptosis [64, 74]. Individual eIF4G isoforms also appear to be at work in* Drosophila* and mouse spermatocytes, where eIF4G homologue,* off-schedule* (eIF4G2), and* Repro8* (eIF4G3), respectively, have been identified as integral regulators of meiotic progression and differentiation [61, 75, 76]. Developing* Drosophila* spermatocytes depleted of eIF4G2 are small in size and accumulate CDK inhibitor protein, RUX, likely as a growth checkpoint before meiotic division [75]. In mouse spermatocytes,* Eif4g3* mutation results in meiotic prophase arrest and the apparent loss of* Hspa2* mRNA translation. HSPA2 is necessary for activation of meiotic prophase kinase CDC2A [76]. These activities define a dynamic system in which the ratio of cap-dependent to cap-independent translation supports cell fates that range from growth to recovery from cellular insult to physiological apoptosis [74, 77].

**Figure 2:**
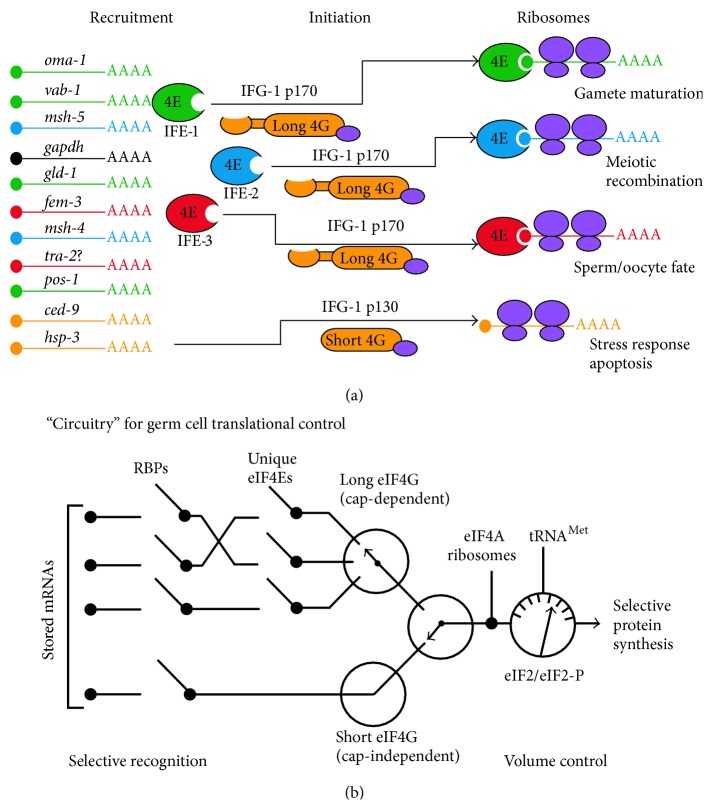
Dynamic models for selective protein synthesis in germ cells. (a) A complex mixed population of mRNAs present in germ cells of various stages is selectively recruited for translation initiation by individual eIF4 isoforms. This positive selection occurs temporally as developing cells require new protein synthesis. Corresponding mRNA becomes derepressed, and the eIF4E-mRNA complex is recruited to the cap-dependent translation initiation complex by the “long” isoform of eIF4G. Other mRNAs are recruited by cap-independent translation initiation via the short eIF4G isoform that lacks the eIF4E binding domain. Episodes of selective mRNA translation by individual eIF4 isoforms drive critical germ cell fate decisions. (b) As new protein synthesis is required for germ cell renewal, growth, and differentiation, one pathway, or circuit, is activated for the translation of a certain population of stored mRNAs. mRNP complexes reach the first “translation on/off” switch at a point where bound RBPs (including eIF4 factors) undergo remodeling that results in mRNA derepression. Derepressed mRNAs following the cap-dependent circuit use a switch involving of one of the eIF4E isoforms. Successfully activating this switch, eIF4E-bound mRNA is recruited to the initiation complex via the long eIF4G. Alternatively derepressed mRNAs following the cap-independent circuit are made available for initiation recruitment via the short eIF4G in an analogous fashion. Both cap-dependent and cap-independent recruited mRNAs then reach a node in which eIF4A and ribosomes must be bound. (Note that in* C. elegans *and* Drosophila* germ cell mRNPs, eIF4A, or a homologous helicase, has been found to be prebound.) The 40S ribosomal subunit brings with it initiator Met-tRNA bound to eIF2. This step constitutes a “rheostat” in the circuit where the volume of protein synthesis can be limited by phosphorylation of eIF2. mRNAs completing this circuit are efficiently decoded into new proteins necessary for discrete germ cell developmental events.

The determinants for specific mRNA binding to eIF4 factors remain unclear. Despite their highly specific ligand binding pockets, all known cap-binding protein eIF4E isoforms have rather low binding affinities for m^7^GTP and no mRNA sequence recognition beyond the first two nucleotides [78–80]. Yet surprisingly, eIF4E isoforms show marked substrate specificity* in vivo* to recruit unique populations of mRNAs. The explanation for this recruitment specificity is twofold. First, eIF4E isoforms are expressed in a tissue-specific fashion in organs that require them, and the constellation of eIF4Es present will therefore differ from tissue to tissue. For example, in* C. elegans* IFE-4 is expressed in the somatic tissues while IFE-1, IFE-3, and IFE-5 are the predominant isoforms expressed in the germline [54]. A cell-type specific isoform can obviously only translate mRNAs to which it has access. However, some eIF4Es coexist in the same cell type, yet they have been shown to translate different mRNAs [63]. This second aspect of mRNA recruitment specificity appears to be due to the fact that each form exists in a different mRNP complex. IFE-1 is bound to an RBP known as PGL-1, which in* C. elegans* oocytes is required for IFE-1's localization with stored mRNAs in P granules [65, 81]. By contrast, IFE-3 is found to be associated with OMA-1 mRNPs in those same oocytes [46]. These two eIF4E isoforms are thus differentiated by the eIF4E binding proteins (4EBPs) with which they interact. Evidence from somatic cell translational control shows that general cap-dependent eIF4E-mediated recruitment of mRNAs is generally inhibited by 4EBPs. Sequestration allows cap-independent initiation to prevail [82, 83]. Growth factor signaling activates mTOR kinase to phosphorylate 4EBP, causing its dissociation from eIF4E and thus restoring cap-dependent protein synthesis. Among the known 4EBPs are several germ cell types that also bind mRNAs, either individually or in complexes. Maskin-CPEB binds and represses eIF4E-bound mRNAs in* Xenopus* oocytes [84]. Following progesterone signaling, maskin dissociates from eIF4E and the mRNA becomes actively recruited for translation initiation via eIF4E. Similarly in* Drosophila* oocytes, Cup is a 4EBP that represses* oskar* translation in an RNP complex with eIF4E [85]. As described above,* C. elegans* PGL-1 is a 4EBP and RBP in germ granules that binds (and presumably represses) only one eIF4E type. These represent a few instances in which RBPs coordinate with eIF4E to prevent the recruitment of mRNAs to eIF4G. Many RBPs bind recognition motifs in mRNA 3′UTRs. As the regulated mRNAs become required, RBP complexes become remodeled (Figure 2(a)) such that eIF4E can associate with eIF4G and recruit the message directly to ribosomes, effectively coordinating the transition from repression to activation [18].

Perhaps the best understood example of molecular events involved in eIF4E regulation in an mRNP complex was described for oocyte maturation in* Xenopus.* Oocyte meiosis is arrested by the translational suppression of* cyclin B* mRNA that contains a cytoplasmic polyadenylation element (CPE) in the 3′-untranslated region [86]. This suppression occurs when the eIF4E-maskin-CPEB (CPE binding factor) complex forms on* cyclin B* mRNA with a short poly(A) tail. Maskin acts as a specialized 4EBP by binding to eIF4E at the eIF4G binding site to occlude its ability to enter the translation initiation complex [19, 84, 87]. Maskin also binds to CPEB to repress the mRNA. To resume oocyte maturation, progesterone signals the activation of Aurora kinase, which phosphorylates CPEB. Active CPEB releases maskin and subsequently recruits CPSF (polyadenylation specificity factor) and poly(A) polymerase (PAP) to the 3′ end of the bound mRNA. The elongated poly(A) tail attracts multiple copies of poly(A)-binding protein (PABP). Both the freed eIF4E and polymerized PABP associate with eIF4G in a productive initiation complex (Figure 1(b)), beginning the synthesis of Cyclin B and driving meiotic maturation. eIF4E's role in regulating cell cycle and proliferation in tumor models, together with its integral role in oocyte meiotic cell cycle progression, has made it a popular therapeutic target in cancer treatment studies and enhanced our understanding of cancer cell translational control [7–12].

Coordinated mRNP repression and derepression are a conserved germ cell strategy in many species from worms to mammals. Mouse prospermatogonia (gonocytes) in the neonatal testes transition into populations of undifferentiated and differentiated spermatogonia. When spermatogonia enter meiosis as spermatocytes, gene transcription is strongly silenced [88, 89]. As in oocytes, stored mRNAs are repressed in mRNP complexes for later protein synthesis [90–92]. One such translationally controlled mRNA encodes the receptor tyrosine kinase* c-Kit*, required for spermatogonial differentiation. Retinoic acid (RA) activates PI3K/AKT/mTOR signaling, which, in turn, phosphorylates 4EBP1. Therefore, activation of eIF4E-mediated initiation correlates with* c-Kit* mRNA recruitment into polysomes for efficient translation [93]. Resulting c-Kit protein is an important meiotic marker for sperm development. Better understood is the translational control of* oskar* mRNA in* Drosophila*, which is necessary for embryonic posterior patterning and germ cell formation. Repression and derepression are both mediated by an eIF4E-Cup-Bru RBP complex on the* oskar* mRNA [57, 62]. During the early oogenesis,* oskar* is transcribed in nurse cells and repressed during its transport to the posterior pole of the oocyte to prevent precocious development [94]. The Bru RBP binds a sequence element in* oskar* 3′UTR as well as eIF4E-Cup complex at the 5′ cap to repress* oskar* translation. Premature translation of* oskar* mRNA prior to localization leads to embryonic patterning defects [62]. The cell polarity established by Oskar is necessary for asymmetrical divisions and body patterning and is governed by several such proteins expressed in a gradient across the cell. The fertilized egg consequently develops organizing centers at both the anterior and posterior poles. The* bicoid* mRNA is localized to the anterior pole. Localized synthesis of Bicoid protein is necessary for head and thorax development. At the posterior pole, by contrast,* nanos* mRNA is localized. When expressed, Nanos protein binds Pumillio to form an mRNP complex that suppresses* hunchback* translation [95]. Nanos and Pumillio have also been implicated in the suppression of* cyclin B* mRNA in early development. Yet, while these RBPs repress mRNAs together in early embryos, they may have different partners in the germline. Nanos is required for proper germ cell migration while Pumillio is necessary for germline stem cell maintenance [96]. Pumillio represses* smaug* mRNA in the embryonic posterior pole. Smaug is itself an RBP that interacts directly with Oskar and prevents* nanos* translation [97]. However, the* smaug* mRNA 3′UTR is also predicted to bind RBPs that promote its translation. One such instance of positive translational control by an RBP involves the highly conserved DAZL protein, which stabilizes mRNAs by promoting PAPB recruitment, circularization of the mRNA, and thus increased translational efficiency in developing germ cells [98]. These anecdotal instances outline a diverse regulatory system in which RBPs and translation initiation factors work together for both negative and positive translational control that drives specific protein synthetic events necessary for germ cell fate decisions.

## 5. The Selective Function of the eIF4 Initiation Complex

Differential recruitment of specific mRNAs by eIF4E and eIF4G isoforms has been studied extensively in* C. elegans* germ cells. Cap-binding eIF4E is present in five isoforms (IFE-1–5) in nematodes. Three forms (IFE-1, IFE-3, and IFE-5) are enriched in or exclusive to the germline, while two isoforms (IFE-2 and IFE-4) are expressed primarily in somatic tissue [54]. Null mutations in individual eIF4E genes have shown that each isoform has a unique subset of mRNAs that it preferentially recruits [65–68, 99]. Other nonregulated mRNAs (like beta-tubulin or GAPDH) appear to be indiscriminate in their choice of eIF4E form. A unique germline eIF4E (IFE-1), for instance, is a key positive translational regulator of multiple steps in sperm and oocyte progression.* ife-1* (−/−) worms are temperature sensitive and sterile due to defective cytokinesis late in spermatogenesis [63]. Hermaphrodites display substantially reduced oocyte growth, maturation, and egg fertilization. Eggs that are successfully fertilized often arrest as early embryos. Follow-up studies showed that IFE-1 promotes germ cell development and preparation for embryogenesis by positively recruiting critical mRNAs for steps in each process (e.g.,* mex-1, oma-1, glp-1, gld-1, pos-1, pal-1, vab-1, rab-7, ran-1, *and* rnp-3*) [63, 100]. Selective translational recruitment by IFE-1 has been demonstrated* in situ* in live worms as mRNAs become activated in a temporal and spatial manner within individual germ cells [100]. Another germ cell eIF4E (IFE-3) is expressed in the same oocyte stages as IFE-1, but its subcellular localization differs. IFE-1, but not IFE-3, colocalizes to germ granules (P granules) via protein-protein binding to PGL-1 [65, 101]. Loss of IFE-3 results in a distinctly different germ cell phenotype.* ife-3* (−/−) animals produce only sperm, even in hermaphrodite adults that should normally switch all germ cell differentiation to oogenesis (Subash and Keiper, unpublished). The strictly spermatogenic fate suggests that the sperm to oocyte switch that normally occurs in late larvae has malfunctioned. This gamete sex switch involves translational control of* tra-2* and* fem-3* mRNAs [102]. Recent observation that IFE-3 interacts with the IFET-1/CGH-1/LARP-1 complex on* fem-3* mRNA suggests another instance of a transitional mRNP complex involving a specific eIF4E [103–105]. Loss of still another eIF4E isoform (IFE-2), which is expressed at very low levels in germ cells, leads to temperature-sensitive meiotic catastrophes. Specifically,* ife-2* (−/−) germ cells have a severe defect in chromosome crossover resolution and repair during meiosis [66]. The repair activities are due to IFE-2-mediated recruitment of key mRNAs (*msh-4/him-14*,* msh-5*) required for proper meiotic chromosome segregation. In just these few examples of distinct roles for three nematode eIF4Es, it is apparent that the translational apparatus itself carries out critically important mRNA selections that alter germ cell fates.

Translational regulation of mRNAs in* C. elegans* germ cells is not limited to the cap-binding activity of eIF4Es. Orthologues of eIF4G (IFG-1), to which IFEs bind to join the ribosome, also exert preferential mRNA recruitment and influence germ cell fate. Two isogenic forms of eIF4G are expressed in worms: a long p170 IFG-1 that has binding sites for IFEs and an N-terminally truncated short p130 IFG-1 that does not (Figures 1(b) and 1(c)). Modest depletion of both p130 and p170 IFG-1, and thus both cap-dependent and cap-independent translation, suppresses the initial expansion of GSCs that occurs in early L2 larval worms as well as the somatic growth and molting of the young worms [64]. Immature worms live nearly a complete lifespan but are unable to grow and appear fully arrested in development. Most interesting, however, are the consequences of altering the balance between the cap-dependent (p170) and cap-independent (p130) IFG-1 activities. These consequences are manifested primarily in the germline. RNAi or genetic depletion of IFG-1 p170 (long eIF4G, Figure 1) alone amplifies the natural proportion of germ cell apoptotic events in differentiating oocytes [71]. The deaths are not spurious collateral damage from constrained protein synthesis. Rather, they require apoptotic signaling through the apoptosome via Apaf-1 (*ced-4*) and caspase (*ced-3*). Germ cell deaths appear to be driven by IFG-1 p130-sustained, cap-independent translation of mRNAs that signal stress, recovery, and eventually apoptosis. Enhanced translation of the chaperone BiP (*hsp-3*) and the apoptotic regulator Bcl-2 (*ced-9*) mRNAs occurs in a background of less efficient translation of many “normal” mRNAs [72]. Overall, the integrated positive contributions of four selective germ cell eIF4Es and two eIF4Gs, in concert with the better-known RBP repressors, lead to a circuitry of translational control that has great latitude for mRNA types and temporal events in germ cell life (Figure 2(a)). There is also considerable evidence from mouse and* Xenopus* oocyte studies for the involvement of eIF2 activity in regulating translation initiation. eIF2 brings the initiator Met-tRNA to the mRNA complex and is subject to phosphorylation by the GCN2 kinase to regulate the volume (overall output) of protein synthesis in late oogenesis and at meiotic maturation [106–111]. This multifaceted mRNA handling system in germ cells at all stages of their development maintains the sophistication of the translation initiation functions of eIF4 and eIF2 factors, in conjugation with repressor RBPs, to carefully govern which, when, and how much of each protein is made (Figure 2(b)). Evidence from unique cases of mRNA translational control observed in divergent animal species has led to a paradigm in which the translation initiation machinery itself acts as integral part of the regulatory pathway at multiple critical transitions in germ cell progression.

Positive translational control of mRNAs is quickly becoming recognized as equally important as repression by RBPs for translational control in developmental contexts. Because this mode of posttranscriptional gene regulation predominates in determining cell fate beginning with GSCs and continuing through early embryonic development, the cooperative nature of eIFs and RBPs will merit further exploration. Key regulatory proteins for both positive and negative mechanisms are highly conserved in all sexually reproducing animal species that have been studied. Disruption of* de novo* protein synthesis changes can lead to severe germ cell deficiencies or aberrant differentiation paths and may contribute to infertility and birth defects. Given the conserved molecular themes in translational control, the interplay between eIFs and RBPs can now be explored in a broad range of animal germ cells to yield principles that should apply to all.
